# Additive effect of 5-HT2C and CB1 receptor blockade on the regulation of sleep–wake cycle

**DOI:** 10.1186/s12868-019-0495-7

**Published:** 2019-03-20

**Authors:** Emese Bogáthy, Noémi Papp, Laszló Tóthfalusi, Szilvia Vas, György Bagdy

**Affiliations:** 1 Department of Pharmacodynamics, Semmelweis University, Budapest, Nagyvárad tér 4, 1089 Hungary; 2 MTA-SE, Neuropsychopharmacology and Neurochemistry Research Group, Budapest, 1089 Hungary; 3 NAP-A-SE, New Antidepressant Target Research Group, Budapest, 1089 Hungary; 4 NAP-2-SE, New Antidepressant Target Research Group, Budapest, 1089 Hungary; 5 Department of Physiology, Development and Neuroscience, University of Cambridge, Cambridge, CB2 3DY UK

**Keywords:** Serotonin 2C receptor, SB-242084, Cannabinoid 1 receptor, AM-251, Sleep, Electroencephalography

## Abstract

**Background:**

Previous data show that serotonin 2C (5-HT_2C_) and cannabinoid 1 (CB_1_) receptors have a role in the modulation of sleep–wake cycle. Namely, antagonists on these receptors promoted wakefulness and inhibited rapid eye movement sleep (REMS) in rodents. The interaction of these receptors are also present in other physiological functions, such as the regulation of appetite. Blockade of 5-HT_2C_ receptors modulat the effect of CB_1_ receptor antagonist, presumably in consecutive or interdependent steps. Here we investigate, whether previous blockade of 5-HT_2C_ receptors can affect CB_1_ receptor functions in the sleep–wake regulation.

**Results:**

Wistar rats were equipped with electroencephalography (EEG) and electromyography (EMG) electrodes. Following the recovery and habituation after surgery, animals were injected intraperitoneally (ip.) with SB-242084, a 5-HT_2C_ receptor antagonist (1.0 mg/kg) at light onset (beginning of passive phase) followed by an injection with AM-251, a CB_1_ receptor antagonist (5.0 or 10.0 mg/kg, ip.) 10 min later. EEG, EMG and motor activity were analyzed for the subsequent 2 h. Both SB-242084 and AM-251 increased the time spent in active wakefulness, while decreased the time spent in non-REMS and REMS stages in the first 2 h of passive phase. In combination, the effect of the agents were additive, furthermore, statistical analysis did not show any interaction between the effects of these drugs in the modulation of vigilance stages.

**Conclusions:**

Our results suggest that 5-HT_2C_ receptor blockade followed by blockade of CB_1_ receptors evoked additive effect on the regulation of sleep–wake pattern.

## Background

The serotonin (5-HT) and the endocannabinoid (eCB) systems show clear interaction in the regulation of various physiological functions, like anxiety and depression [1–3], coping with stress [4], fear extinction [5] and in the regulation of appetite [6, 7]. The 5-HT system has a well-known role in the sleep–wake regulation as well. Serotonergic neurons fire most actively during wakefulness, decrease their activity rate during non rapid eye movement sleep (non-REMS) and fall silent during rapid eye movement sleep (REMS) [8]. Several data support that the eCBs and the cannabinoid 1 (CB_1_) receptors also affect the circadian rhythmicity and the sleep–wake cycle. The eCBs might participate in the sleep promotion by increasing the time spent in non-REMS and REMS, while reducing wakefulness [9]. At the same time, considering the connection between the 5-HT and the eCB systems in the sleep–wake regulation, only a few studies can be found in the literature. The increase in the time spent in slow wave sleep (SWS) by oleamide (a cannabimimetic molecule) was prevented by 5-HT reuptake inhibitors such as fluoxetine or fenfluramine, but also by agonists of the 5-HT_1A_ receptors [10]. Oleamide has also been reported to potentiate the action of 5-HT on 5-HT_2C_ receptors expressed by Xenopus oocytes [11].

The 5-HT_2C_ and CB_1_ receptors are widely distributed in sleep-modulating areas of the brain, frequently located on local inhibitory gamma-aminobutyric acidergic (GABAergic) interneurons and glutamatergic neurons [12–15]. The GABA release, caused by the activation of 5-HT_2C_ receptors consequently evokes inhibitory effect on monoaminergic cell groups [13, 16]. Accordingly, the role of 5-HT_2C_ receptors has been demonstrated in the sleep–wake regulation as well. Administration of the 5-HT_2C_ agonists, RO 60-0175 and RO 60-0332 increased wakefulness and decreased REMS [17]. In line with this, mice lacking 5-HT_2C_ receptors had greater amounts of wakefulness and spent significantly less time in non-REMS compared to wild-type controls [18]. Injections of ritanserin and ketanserin, possessing 5-HT_2A/2C_ receptor antagonist properties, induced a significant increase in SWS and a reduction of both REMS and wakefulness in rats [19–23]. However, our previous data show that SB-242084, a highly selective 5-HT_2C_ receptor antagonist, promotes wakefulness while decreases both deep slow wave sleep (SWS-2) and REMS [24, 25].

The role of eCBs in the promotion and maintenance of sleep have also been supported by genetic studies, namely CB_1_ receptor knockout mice spent more time in wakefulness compared to their wild-type littermates [26, 27]. The CB_1_ receptor antagonists SR141716a (rimonabant) and AM-251 have been reported to increase wakefulness, reduce both non-REMS and REMS in monotherapy [28, 29], moreover, could block sleep–wake alterations caused by eCBs [30].

Exploration of the eCB system is still in the focus of research. Up-regulation of the eCB system has been found in various disorders like obesity, metabolic disorder, osteoporosis, hyperalgesia, intestinal inflammation, and in certain cases of impaired fertility in women [14]. Thus, investigating the effects of CB_1_ receptor blockade and its interaction with another neurochemical pathways may open a way for new therapeutic application of these drugs.

In behavioral studies, more specific interactions have been described between the CB_1_ and 5-HT_2C_ receptors. Soria-Gomez et al. [31] have shown that hypophagia induced by oleamide and AM-251 has been blocked by SB-242084. Based on the above mentioned findings, we aimed to investigate how previous 5-HT_2C_ receptor blockade modifies the effect of a CB_1_ receptor antagonist on the pattern of sleep–wake cycle.

## Methods

### Animal maintenance

All animal experiments and housing conditions were carried out in accordance with the EU Directive 2010/63/EU and the National Institutes of Health “Principles of Laboratory Animal Care” (NIH Publications No. 85-23, revised 1985), as well as specific national laws (the Hungarian Governmental Regulations on animal studies 40/2013). The experiments were approved by the National Scientific Ethical Committee on Animal Experimentation. Male, drug and test naïve Wistar rats were purchased from Animal Facility (Semmelweis University, Budapest, Hungary). Rats (8 weeks old), weighing 300–330 g at surgery, were kept under controlled environmental conditions (temperature at 21 ± 1 °C and a 12 h light–dark cycle starting at 10:00 A.M.). Rats were kept three per cage before surgery. Food and water were available ad libitum during the whole experiment. All efforts were made to minimize pain and suffering of the rats. Rats were euthanized with an overdose of anesthetic (halothane) when the experimental procedure was finished.

### Surgery

Rats were chronically equipped with electroencephalographic (EEG) and electromyographic (EMG) electrodes, as described earlier [32]. Briefly, stainless steel screw electrodes were implanted epidurally over the left frontal cortex (2.0 mm lateral and 2.0 anterior mm to bregma) and left parietal cortex (2.0 mm lateral and 2.0 anterior mm to lambda) for frontoparietal EEG recordings. The ground electrode was placed over the cerebellum. EMG electrodes (stainless steel spring electrodes embedded in silicon rubber; Plastics One Inc., Roanoke, VA, USA) were placed into muscles of the neck. Surgery was performed under halothane (2%) anaesthesia (Fluotec 3 vaporizer) using a Kopf stereotaxic instrument.

### Drug administration

SB-242084 [SB, 6-chloro-5-methyl-1-[2-(2-methylpyrid-3-yloxy)-pyrid-5-yl carbamoyl] indoline] and AM-251 [AM, N-(Piperidin-1-yl)-5-(4-iodophenyl)-1-(2,4- dichlorophenyl)-4-methyl-1H-pyrazole-3-carboxamide] were purchased from Tocris Cookson (Bristol, UK). Both compounds were dissolved in vehicle (veh) consisted of 70% PBS (phosphate buffered saline, pH = 7.4), 20% dimethylsulfoxide and 10% Tween 80. All rats were treated with a first (veh or 1 mg/kg SB) and a second [veh or 5 mg/kg AM (AM D5) or 10 mg/kg AM (AM D10)] intraperitoneal (ip.) injections in 1 ml/kg volume, with 10 min difference between the injections. Animals (n = 6) were treated with the following treatments, in crossover design: veh + veh, veh + AM D5, veh + AM D10, SB + veh, SB + AM D5, SB + AM D10.

### EEG recording

After surgery, rats were kept in a square, glass recording chamber separately. After a 7-day-long recovery period, rats were attached to the EEG system by a flexible recording cable and an electric swivel, fixed above the cages, permitting free movement of the animals. In order to habituate the animals to the recording conditions, rats were attached to the EEG system 7 days before starting the treatments, and were kept connected to the system during the whole experiment. To assess motor activity, electromagnetic transducers were used, in which potentials were generated by movements of the recording cable, as described earlier [32]. EEG, EMG and motility were recorded during a 24-h long period, starting at light onset (10:00 A.M.). Rats were undisturbed throughout the recordings and had free access to standard rodent chow and tap water. The signals were amplified (amplification factors approximately 5000 for EEG and motor activity, 20000 for EMG), conditioned by analogue filters (Coulburn Lablinc System, USA; filtering below 0.50 Hz and above 100 Hz at 6 dB/octave), and subjected to analogue to digital conversion with a sampling rate of 256 Hz. Data were stored on a computer for further analysis.

### Sleep scoring

The vigilance stages were classified automatically by SleepSign for Animal sleep analysis software (Kissei Comtec America, Inc., USA) followed by visual assessment for 4-s periods over 2 h, as described earlier [32]. The visual assessment and scoring were performed by an observer blind to the treatments. The vigilance stages scored in the present study were the following: active wakefulness (AW), the EEG is characterized by low-amplitude activity at beta (14–30 Hz) and alpha (8–13 Hz) frequencies accompanied by high EMG and intense motor activity; passive wakefulness (PW), the EEG is characterized by low-amplitude activity at beta (14–30 Hz) and alpha (8–13 Hz) frequencies accompanied by high EMG activity but minimal or no motor activity; light slow wave sleep (SWS-1), high-voltage slow cortical waves (0.5–4 Hz) were interrupted by spindles (6–15 Hz) accompanied by reduced EMG and no motor activity; deep slow wave sleep (SWS-2), there were continuous high-amplitude slow cortical waves (0.5–4 Hz) with reduced EMG and no motor activity; intermediate stage of sleep (IS), a brief stage just prior to REMS and sometimes just after it, characterized by unusual association of high-amplitude spindles (mean 12.5 Hz) and low-frequency (mean 5.4 Hz) theta rhythm; rapid eye movement sleep (REMS), low-amplitude and high-frequency EEG activity with regular theta waves (5–9 Hz) were accompanied by silent EMG and motor activity with occasional twitching. SWS-1 and SWS-2 sleep stages together create the non-REMS stage. The following parameters were calculated (1–2 h following treatments): total time spent in each vigilance stage (AW, PW, SWS-1, SWS-2, REMS, IS); the occurrence of the first REMS period (REMS latency). An episode of any vigilance stage was defined as an item lasting at least 4 s and not interrupted by any other vigilance stage for longer than 4 s.

### Statistical analysis

For statistical analysis STATISTICA 7.0 (Statsoft Inc., Tulsa, OK, USA) software was used. Data were analyzed by two alternative statistical approaches, the conclusions were drawn based on these two methods. Sleep parameters of the treatment groups were evaluated by two-way analysis of variance (ANOVA) with two factors: pretreatment [veh or SB] and treatment [veh or AM DX (X = 5 or 10 mg/kg dose)] and their interaction. We also performed one-way ANOVA tests in each vigilance stage including the 6 treatment groups. In case of significant main effects of the one-way ANOVA tests, the difference between the groups was investigated by Dunnett’s post hoc analysis (indicated on the figures). In contrast to two-way ANOVA, the post hoc methods compare the means of groups, with the special case of Dunnett’s test, that compare all treated groups to the control one. The two methods are complementary to each other. The two-way ANOVA method is more powerful but the Dunnett’s method is based on fewer statistical assumptions. In both statistical tests p values less than 0.05 (p < 0.05) were defined as statistically significant. Data on the figures are presented as mean ± SEM.

## Results

### SB-242084 and AM-251 showed additive active wake-promoting effect

**Fig. 1:**
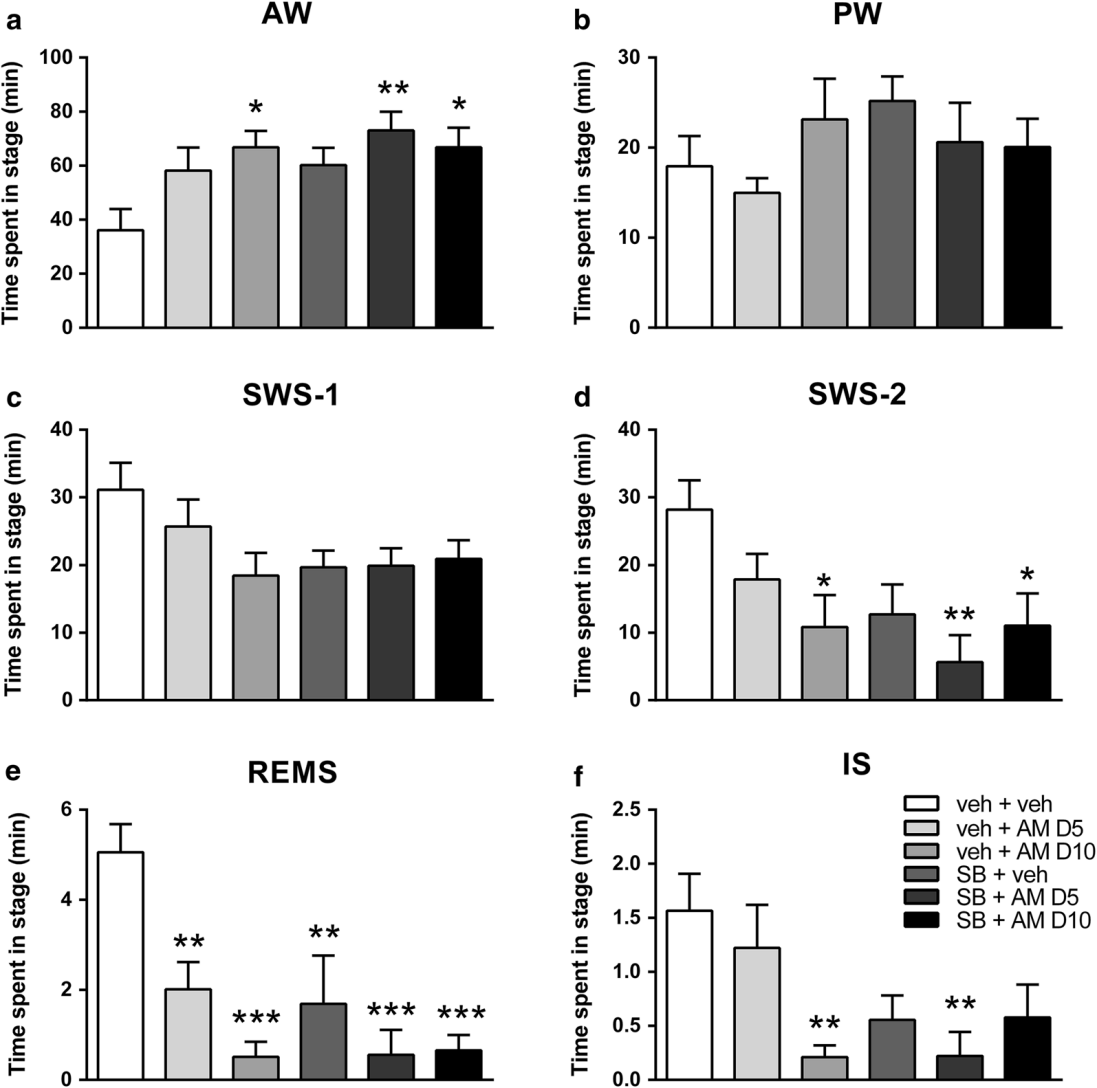
The effects of SB-242084 (SB, 1 mg/kg ip.) and AM-251 (AM D5, 5 mg/kg ip. and AM D10, 10 mg/kg ip.) treatments on the time spent in **a** active wakefulness (AW); **b** passive wakefulness (PW); **c** light slow wave sleep (SWS-1); **d** deep slow wave sleep (SWS-2); **e** rapid eye movement sleep (REMS); **f** intermediate stage of sleep (IS) in the summarized first 2 h of the passive phase. Data are presented as mean ± SEM of the 6 animals. *p < 0.05, **p < 0.01, ***p < 0.001 compared to the veh + veh control group (Dunnett’s post hoc comparison)

With regard to wakefulness, SB-242084 and AM-251 treatments showed additive effect to increase the time spent in AW. However, post hoc analysis did not reveal significant effect of SB or AM D5 in mono-treatments, compared to the veh + veh control group. The AW-promoting effects of the drugs were statistically significant when administered simultaneously. The effect of AM D10 was significant in mono-treatment and in combination with SB as well (Fig. 1a). Two-way ANOVA showed significant effect of both SB and AM D5 (*F*_1,20_ = 6.777; *p *= 0.0170 and *F*_1,20_ = 5.482; *p *= 0.0297, respectively) on the time spent in AW, but we did not find interaction between the two agents. Regarding the combination of SB with the higher AM dose, two-way ANOVA showed a tendency of SB to increase AW, a significant effect of AM D10 (*F*_1,20_ = 3.002; *p *= 0.0985 and *F*_1,20_ = 7.263; *p *= 0.0139, respectively) on the time spent in AW and a trend in the interaction effect (*F*_1,20_ = 3.025; *p *= 0.0974).

Regarding PW, we did not find any significant effect of SB, AM D5 or AM D10, or their combination in post hoc analysis or with two-way ANOVA statistics (Fig. 1b).

### SB-242084 and AM-251 caused additive reduction in slow wave sleep

In SWS-2 stage, the post hoc analysis after significant one-way ANOVA showed a trend effect of SB (*p *= 0.0683), although AM D5 treatment did not cause any significant effect. However, AM D10 mono-treatment showed a significant decrease in the time spent in SWS-2 compared to the veh + veh group. At the same time, the combination of SB with AM D5 or AM D10 doses showed a significant decrease in time spent in SWS-2 in both cases. Interestingly, this effect seemed to be stronger in case of the SB + AM D5 combination (Fig. 1d). Two-way ANOVA statistics revealed a significant effect of both the pretreatment with SB (*F*_1,20_ = 11.25; *p *= 0.0032), and the treatment with AM D5 (*F*_1,20_ = 4.402; *p *= 0.0488). However, we did not find any interaction between the treatments (*F*_1,20_ = 0.1509; *p *= 0.7018). AM D10 treatment also showed a trend effect (*F*_1,20_ = 4.305; *p *= 0.0511) in two-way ANOVA, but no interaction was found between the SB and AM D10 treatments.

Regarding SWS-1, SB significantly decreased the time spent in this stage (pretreatment effect: *F*_1,20_ = 6.648; *p *= 0.0179), but we did not find any significant treatment effect of AM D5 or AM D10 (Fig. 1c).

Taken together, in monotherapy the time spent in SWS-2 was decreased by SB, AM D5 and AM D10 as well, while the time spent in SWS-1 was reduced by SB only. In combination, SB and AM showed a significant decrese in SWS-2 by post hoc results, compared to control. Both AM D5 and AM D10 treatments were capable to increase the effect of SB, suggesting additive effect of the drugs on reducing SWS-2 only.

### SB-242084 and AM-251 suppressed REMS and IS

Regarding the post hoc tests after significant one-way ANOVA, SB, AM D5 and AM D10 showed significant REMS-reducing effect in both mono-treatment and in combinations (Fig. 1e). According to two-way ANOVA, both SB and AM D5 treatments significantly decreased the time spent in REMS (*F*_1,20_ = 10.48; *p *= 0.0041 and *F*_1,20_ = 7.868; *p *= 0.0109, respectively), but we did not find any interaction effect (*F*_1,20_ = 1.646; *p *= 0.2141). Similarly, significant pretreatment and treatment effects were seen in two-way ANOVA, when combining SB with AM D10 (*F*_1,20_ = 5.866; *p *= 0.0251 and *F*_1,20_ = 17.58; *p *= 0.0004, respectively) and an interaction effect (*F*_1,20_ = 6.964; *p *= 0.0157) as well.

Regarding REMS latency, two-way ANOVA revealed that, both SB and AM D5 treatment significantly increased REMS latency (SB effect: *F*_1,19_ = 12.35; *p *= 0.0023; AM effect: *F*_1,19_ = 9.045; *p *= 0.0072). At the same time, in the SB + AM D10 combination, two-way ANOVA showed a significant treatment effect of AM D10 (*F*_1,15_ = 6.265; *p *= 0.0244), moreover, an interaction between the two agents (*F*_1,15_ = 9.732; *p *= 0.0070).

Regarding the time spent in IS, post hoc tests after one-way ANOVA showed, that AM D10 reduced IS significantly compared to veh + veh control group, while AM D5 produced a similar reduction when combined with SB (Fig. 1f). Two-way ANOVA showed a significant effect of SB in combination with AM D5 (*F*_1,20_ = 10.82; *p *= 0.0037). However, in the SB + AM D10 combination, AM D10 showed significant effect alone and an interaction effect as well (*F*_1,20_ = 6.532; *p *= 0.0188 and *F*_1,20_ = 6.975; *p *= 0.0157, respectively).

To sum up, both SB and AM in both doses tended to decrease REMS. However, while the effect of AM D5 seemed to be added to the REM-suppressing effect of SB, AM D10 could not produce this additive effect, despite decresing REMS alone. The cause of this can be that the maximal REMS-decreasing effect was already reached by AM D10 in mono-treatment, thus, further enhancement of this effect was not possible by the combined treatment.

## Discussion

Our study confirms, in agreement with previous findings, that the combination of 5-HT_2C_ and CB_1_ receptor antagonists increases wakefulness, while decreases the time spent in non-REMS and REMS stages as a result of an additive effect of the agents. Based on two-way ANOVA, both SB and AM influenced AW, SWS-2, REMS and IS parameters and SB modulates SWS-1 too. The effect of AM was different in D5 and D10 doses. In 10 mg/kg dose AM increased AW, and decreased the time spent in SWS-2, REMS and IS, however, when applied in 5 mg/kg it reduced REMS only. In combination with SB, the effect of AM D5 and D10 was similar, increasing the time spent in AW, while reducing SWS-2 and REMS. The additive effect of SB and AM was obvious in the SB + AM D5 combination. However, based on interaction effects of the SB + AM D10 combination in the time spent in AW and SWS-2 parameters, the additive effect was weaker in this combination.

Neurochemical and electrophysiological studies have found that the role of 5-HT in the sleep–wake regulation is promoting wakefulness and inhibiting REMS [33, 34]. Regarding 5-HT_2C_ receptors, their mRNA has been found in the cholinergic pedunculopontine and laterodorsal tegmental nuclei (PPT/LDT) [12]. These neurons fire most rapidly during wakefulness and REMS, and most slowly during non-REMS [35]. The 5-HT_2C_ receptors have been found in the dorsal raphe nuclei (DRN) as well [13] and are expressed mainly by inhibitory GABAergic interneurons, but also by excitatory glutamatergic neurons [36]. The exact mechanism of the elevation of the amount of wakefulness and the suppression of both non-REMS and REMS caused by the blockade of 5-HT_2C_ receptors is still unclear, but these effects may be partially mediated by the suppressed activity of GABAergic neurons in the DRN. This decrease in the inhibitory influence of DRN may lead to an increase in the monoaminergic cell firing, consequently inducing an inhibition on the PPT/LDT neurons.

The role of eCBs has also been demonstrated in the regulation of the sleep–wake cycle, based on the finding that these bioactive lipids reduced wakefulness and promoted non-REMS and REMS [9]. In line with this, the CB_1_ receptor antagonists SR141716a and AM-251 presented the opposite effects, promoting wakefulness and decreasing both non-REMS and REMS activity [28, 29]. Intracerebroventricular administration of the eCB anandamide (ANA) during the lights-on period caused diminution in wakefulness, while increased non-REMS and REMS in rats. This effect was more evident when ANA was injected directly into the PPT nucleus, and SR141716a prevented this effect [37, 38]. Experimental evidence indicates that the activation of the CB_1_ receptors promotes the release of acetylcholine (ACh) in cortical and hippocampal areas [39]. Therefore, the blockade of CB_1_ receptors in local GABAergic and glutamatergic networks might influence the release of ACh in the PPT/LDT and in the basal forebrain inducing wakefulness and reducing sleep.

With regard to the regulation of REMS by cannabinoids, infusion of 2-arachidonoylglycerol (2-AG) into the lateral hypothalamus caused a massive increase of REMS, while AM-251 was able to block this effect [40]. In the hypothalamic regulation of the sleep–wake cycle, WIN 55,212-2, a potent CB_1_ receptor agonist has been shown to depolarize melanin-concentrating hormone (MCH) neurons in the hypothalamus in vitro [41]. In line with this, 2-AG activates these neurons in vivo, thereby increasing REMS [42]. MCH neurons promote non-REMS and REMS, by showing their maximal firing rate during REMS, moderately firing during non-REMS, and being almost silent during wakefulness [43]. These findings suggest that eCBs also influence the hypothalamic regulation of sleep acting on MCH neurons. Correlation between the amount of REMS and neuronal (Fos) activation of the MCH neurons during rebound sleep after selective REMS deprivation has also been demonstrated by Kitka et al. [44]. In rats, SR141716a has also been shown to prevent REMS rebound following selective REMS deprivation [45].

In our study, SB with the lower dose of AM (5 mg/kg) caused a clear additive effect in AW, SWS-2 and REMS parameters, but these effects were weaker when SB was combined with AM D10. This phenomenon may be due to various reason. Several findings suggested a dose-dependent pharmacological effect of WIN55,212-2 on the CB_1_ receptors of inhibitory and excitatory neurons in the hippocampus of mice, namely the inhibitory synaptic transmission was more sensitive to the effect of cannabinoid receptor modulator, than the excitatory neurotransmission [46, 47]. Another potential reason can be, that, in terms of CB_1_/CB_2_ receptor selectivity, AM-251 similarly to its structural analog SR141716a, is a CB_1_ receptor selective compound in nanomolar concentration, but in higher doses the effect is not CB_1_ specific. Binding experiments showed, that in micromolar concentration, SR141716a and AM-251 can interact on both transient receptor potential vanilloid type 1 and adenosine A1 receptors [48–51]. Thus, the effect of the higher dose of AM-251 on other cell types or receptors might explains, why this dose attenuated the additive effect. In line with our findings, there is another example in the literature, where synergistic or additive effects of CB_1_ and 5-HT_2C_ receptor functions showed dependency on the dose-ratios of the applied drugs. Ward et al. have measured the effect of SR141716a, *meta*-chlorophenylpiperazine (mCPP, a 5-HT_2C_ receptor agonist) and their combination on motivation to consume a palatable drink in mice. When combined in 1:1 and 2:1 dose ratios, SR and mCPP produced significant synergistic effect, while in 3:1 ratio their interaction led to additive effect in attenuation of motivation to consume palatable drink [6].

Although, the additive effect of SB treatment with the higher dose of AM (10 mg/kg) was weaker, AM D10, similarly to AM D5 exerted its effect after SB-242084 treatment as well. Therefore, we can conclude that although the two drugs can produce additive interaction in a given dose, they might also act independently from each other. This is in agreement with literature data (see above) reporting that both SB and AM evoke their sleep–wake modulating effect by influencing PPT/LDT neurons, however, can also exert their effect on different ways. SB modulates DRN neurons in the brainstem leading to the inhibition of PPT/LDT neurons firing, whereas AM influences local GABAergic and glutamatergic networks within the PPT/LDT exerting a more direct effect on these neurons. Luppi et al. [52] have also emphasized the role of local GABAergic and glutamatergic networks over the aminergic-cholinerg projections in REMS regulation. AM modulates the hypothalamic MCH neurons as well, but until nowadays no data is available investigating the effect of SB on this neuron population. Thus, the MCH neuron population might be the other target on which AM can exert its effect independently from SB treatment.

## Conclusion

Taken together, blockade of the 5-HT_2C_ and CB_1_ receptors influences the sleep–wake pattern in a similar way by inducing wakefulness and suppressing both non-REMS and REMS. Our findings reinforce the role of eCB and serotonergic system in the regulation of sleep–wake behavior. These data also suggest that 5-HT_2C_ receptor blockade followed by blockade of CB_1_ receptors evoke additive effects on the regulation of sleep–wake pattern that may provide additional information regarding their interaction in the central nervous system.

As the drugs were administered intraperitoneally, and not into specific nuclei of the brain involved in regulation of vigilance, we cannot exclude the influence of several other factors on their interaction in the regulation of sleep–wake cycle.

## Abbreviations

2-AG: 2-arachidonoylglycerol
5-HT: serotonin
5-HT_1A_ receptor: serotonin 1A receptor
5-HT_2C_ receptor: serotonin 2C receptor
ACh: acetylcholine
AM D10: 10 mg/kg dose of AM-251
AM D5: 5 mg/kg dose of AM-251
AM: AM-251
ANA: anandamide
AW: active wakefulness
CB_1_ receptor: cannabinoid 1 receptor
DRN: dorsal raphe nuclei
eCB: endocannabinoid
EEG: electroencephalography
EMG: electromyography
GABAergic: gamma-aminobutyric acidergic
ip.: intraperitoneally
IS: intermediate stage of sleep
MCH: melanin-concentrating hormone
mCPP: *meta*-chlorophenylpiperazine
non-REMS: non rapid eye movement sleep
PPT/LDT: pedunculopontine and laterodorsal tegmental nuclei
PW: passive wakefulness
REMS: rapid eye movement sleep
SB: SB-242084
SWS: slow wave sleep
SWS-1: light slow wave sleep
SWS-2: deep slow wave sleep
